# Bentonite and Biochar Mitigate Pb Toxicity in *Pisum sativum* by Reducing Plant Oxidative Stress and Pb Translocation

**DOI:** 10.3390/plants8120571

**Published:** 2019-12-05

**Authors:** Muhammad Zulqurnain Haider, Sabir Hussain, Pia Muhammad Adnan Ramzani, Mutahar Iqbal, Muhammad Iqbal, Tanvir Shahzad, Maryam Fatima, Shahbaz Ali Khan, Imran Khan, Muhammad Shahid, Muhammad Ibrahim, Hafiz Syed Tanzeem Ull Haq, Faisal Mahmood

**Affiliations:** 1 Department of Botany, Government College University, Faisalabad 38000, Pakistan; drmzhaider@gcuf.edu.pk; 2 Department of Environmental Sciences and Engineering, Government College University, Faisalabad 38000, Pakistan; Sabir.hussain@gcuf.edu.pk (S.H.); mutahariqbal70@gmail.com (M.I.); iqbal.farhad@gmx.at (M.I.); tanvirshahzad@gcuf.edu.pk (T.S.); mfvirgo000@gmail.com (M.F.); shahbaz_2010@live.com (S.A.K.); ebrahem.m@gmail.com (M.I.); tanzeem_syed@yahoo.com (H.S.T.U.H.); 3 Cholistan Institute of Desert Studies, The Islamia University of Bahawalpur, Bahawalpur 63100, Pakistan; dr.piamuhammad@iub.edu.pk; 4 Department of Agronomy, University of Agriculture, Faisalabad 38040, Pakistan; agronomist786@hotmail.com; 5 Department of Bioinformatics and Biotechnology, Government College University, Faisalabad 38000, Pakistan; mshahid@gcuf.edu.pk

**Keywords:** lead pollution, antioxidants, bentonite, translocation factor, grain biochemistry, biochar, oxidative stress

## Abstract

Lead (Pb)-polluted soils pose a serious threat to human health, particularly by transmitting this heavy metal to the food chain via the crops grown on them. The application of novel amendments in Pb-polluted soils can significantly reduce this problem. In this research, we report the effects of various organic and inorganic amendments i.e., bentonite (BN), biochar (BR), lignin (LN), magnesium potassium phosphate cement (CM) and iron hydroxyl phosphate (FeHP), on the Pb bioavailability in Pb-polluted soil, upon Pb distribution in shoots, roots, grain, the translocation factor (TF) and the bioconcentration factor (BCF) of Pb in pea (*Pisum sativum* L.) grain. Furthermore, effects of the said amendments on the plant parameters, as well as grain biochemistry and nutritional quality, were also assessed. Lead pollution significantly elevated Pb concentrations in roots, shoots and grain, as well as the grain TF and BCF of Pb, while reducing the nutritional quality and biochemistry of grain, plant height, relative water content (RWC), chlorophyll contents (chl a and chl b) and the dry weight (DW) of shoot, root and grain. The lowest Pb distribution in shoots, roots and grain were found with BN, FeHP and CM, compared to our control. Likewise, the BN, FeHP and CM significantly lowered the TF and BCF values of Pb in the order FeHP > CM > BN. Similarly, the highest increase in plant height, shoot, root and grain DW, RWC, chl a and chl b contents, grain biochemistry and the micronutrient concentrations, were recorded with BR amendment. Biochar also reduced grain polyphenols as well as plant oxidative stress. Given that the BR and BN amendments gave the best results, we propose to explore their potential synergistic effect to reduce Pb toxicity by using them together in future research.

## 1. Introduction

Urbanization and industrialization has led to the contamination of world soils with heavy metals [1]. Lead (Pb) is an important pollutant in soil among other heavy metals. It can enter into soils through the disposal of effluents from industries like batteries and paints, mining and smelting, and the burning of fossil fuels, i.e., coal and leaded gasoline [2,3]. Similarly, several natural sources causing soil pollution with Pb are the weathering of rocks, volcanic eruption, forest fires and sea sprays [4]. 

Lead pollution has an adverse effect on humans, especially the health of teenagers. Consumption of food grown within Pb-contaminated soil may cause neurological effects and cognitive disorders [5,6]. Soil pollution with Pb is not only threatening human health, it is also damaging the environment by polluting the ground and surface water resources [6]. 

Given the disadvantages of traditional mitigation practices deployed for heavy metals-polluted soils [7,8], gentle remediation practices like phytoremediation and in-situ immobilization are being promoted. Phytoremediation is a process in which plants are used in combination with suitable agronomic practices to remove heavy metals from the environment, or at least mitigate their toxicity [9]. It involves the use of many approaches individually or in combination. For instance, phytoextraction involves the uptake of toxic metals by plants in their shoots, whereas in phytostabilization, the mobility of pollutants in soils is restricted by using suitable plant cover on contaminated sites [10]. The phytoextraction of heavy metals can be stimulated by using different organic chelants like ethylenediaminetetraacetic acid (EDTA), ethylenediamine-N,N′-disuccinic acid (EDDS) etc., which solubilize heavy metals in the rhizosphere, thereby enhancing their uptake by the plants. However, these chelating agents are slowly biodegradable in nature, and cause ground water contamination [11]. Similar to phytoextraction, phytostabilization can also be enhanced by amending soils with suitable materials that can immobilize metals in the presence of selected plant species. Organic amendments can immobilize certain heavy metals through enhanced organo-metal complexes, chemisorption, ion exchange, complexation and adsorption. Moreover, increase in pH as the result of amendments may also immobilize heavy metals [9].

The recent trend to use magnesium potassium phosphate cement (CM) for Pb immobilization in Pb-polluted soil is more advantageous than ordinary Portland cement (OPC) [6]. The CM converts Pb into highly insoluble pyromorphite (Pb_5_(PO_4_)_3_X, X = Cl^−^, OH^−^, F^−^) and Pb-phosphate (Pb_3_(PO_4_)_2_) while OPC makes Pb(OH)_2_ in a Pb-rich environment. Compared to the Pb(OH)_2_ that has a comparatively high solubility [solubility product constant (Ksp) ≅ 10^−4^], the pyromorphite and Pb-phosphate have much lower solubility (Ksp ≅ 10^−60^–10^−85^ and 10^−6^, respectively). Furthermore, CM has vital properties like fast-setting, high early strength and resistance to soil alkaline or acidic conditions, which all make it an excellent Pb immobilizing agent compared to OPC in Pb-polluted soils [6,12]. Biochar (BR) is prepared by the pyrolyzing organic waste under oxygen-limited conditions [13]. It, when derived from alkaline feedstocks, raises the soil pH similar to liming materials. Moreover, it has a large surface area and high sorption capacity that enable it to effectively lessen the Pb bioavailability in this Pb-polluted soil [3,13,14]. Lignin (LN), a waste product of the paper industry, has abundant oxygen-containing groups, carboxyl, lactonic and phenolic hydroxyl groups [15,16]. Due to these functional groups, LN has been widely used as an effective amendment for the immobilization of Pb in Pb-polluted water and soil [15,16]. Bentonite (BN), an expandable clay mainly comprised of montmorillonite, has high durable negative charges and large definite surface area [7]. Numerous studies have reported that amending metal-polluted soils with BN has significantly reduced the bioavailability of a variety of metals, especially Pb [1,5,7]. Likewise, iron hydroxyl phosphate (FeHP) forms stable metal-phosphate precipitates e.g., Pb_5_(PO_4_)_3_ (OH, F, Cl) in Pb-polluted soils [17].

A lot of research in the past few decades has been carried out on the assessment of various organic and inorganic amendments to reduce the Pb bioavailability in Pb-polluted soils and Pb uptake by different crops. However, there exists no research on the efficacy of different cost-effective and innovative amendments on reducing Pb distribution in pea grain, plant oxidative stress and any improvement in the grain biochemistry and nutritional value and plant agronomic and biophysical traits grown on Pb-polluted soil. Therefore, the key objectives of this research were to (i) evaluate the efficacy of BN, BR, LN, CM and FeHP to reduce Pb bioavailability in Pb-polluted soil, (ii) evaluating the positive effects of Pb immobilization on pea grain quality, productivity and Pb translocation in grain and (iii) to determine the level of Pb uptake and changes in plant oxidative stress and antioxidant defense machinery in response to the said amendments.

## 2. Results

### 2.1. Pb Allocation in Plant Parts and Soil

**Figure 1:**
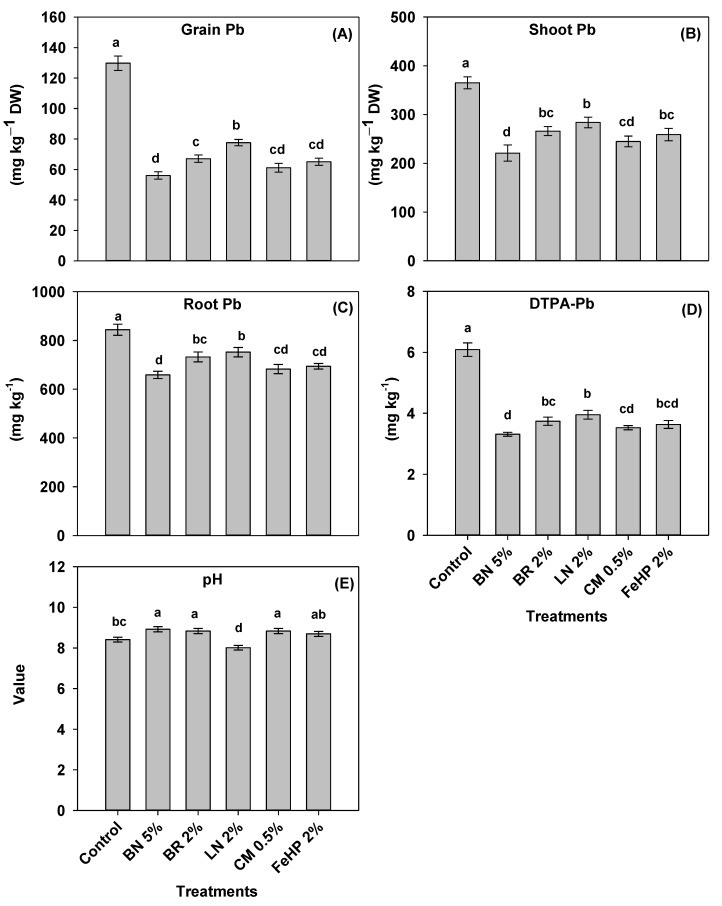
Effects of amendments in Pb-polluted soil on the concentrations of Pb in pea grain (**A**), in the shoot (**B**), the root (**C**) and diethylenetriaminepentaacetic acid (DTPA)-extractable fraction (**D**), as well as soil pH after plant harvest (**E**). Values are the means of three replicates, the error bars represent the standard error of means and the lower case alphabets indicate significant differences (*p* ≤ 0.05) among treatments based on one way Analysis of Variance (ANOVA) (using LSD test, *p* = 0.05 at *df* = 5 and *n* = 3). DW: dry weight; BN: bentonite, BR: biochar; LN: lignin; CM: magnesium potassium phosphate cement; FeHP: iron hydroxyl phosphate.

The data regarding the concentrations of Pb in grain, shoots and roots were in the range of 56.0 to 129.8, 220.9 to 365.1 and 658.6 to 843.8 mg kg^−1^ DW, respectively. The Pb concentration was 3.31 to 6.09 mg kg^−1^ soil in the DTPA extract (Figure 1). Amending the Pb-polluted soil with BN 5%, BR 2%, LN 2%, CM 0.5% and FeHP 2% significantly reduced the Pb concentrations in grain, shoots, roots and DTPA extract, compared to the control. In the BN 5%, CM 0.5% and FeHP 2% treatments, the Pb concentrations in grain were reduced by 57%, 53% and 50%; in roots by 22%, 19% and 18% and in DTPA extract by 46%, 42% and 40%, respectively. Likewise, the BN 5% and CM 0.5% treatments showed the highest reduction in the concentrations of Pb in shoots by 39% and 33%, respectively (Figure 1).

The TF values for Pb were in the range 0.34–0.43 in all treatments (Table 1). All treatments significantly reduced the TF values for Pb when compared to control. The BN 5%, CM 0.5%, and FeHP 2% showed the highest significant decrease in TF values for Pb, compared to control. Overall, the TF values for Pb among various treatments was in the following order: Control > LN 2% > BR 2% > FeHP 2% > CM 0.5% > BN 5%. Similarly, the BCF values for Pb for all treatments were in the range 0.22–0.37 (Table 1). Results revealed that all treatments were able to significantly reduce the BCF values for Pb, compared to control. 

In this context, the BN 5%, FeHP 2% and CM 0.5% treatments exhibited the highest significant reduction in BCF values for Pb, compared to control. The order of reduction in the BCF values for Pb was Control > LN 2% > BR 2% > FeHP 2% > CM 0.5% > BN 5%.

Soil pH after plant harvest ranged from 8.01 to 8.92, with the highest pH values found in BR 2%, CM 0.5% and BN 5%, compared to control. Likewise, the lowest value of pH was found in the LN 2% treatment, compared to control (Figure 1).

### 2.2. Agronomic, Photosynthetic and Biophysical Parameters of Pea Plant as Influenced by Amendments

The obtained data for plant height, shoot, root and grain DW were in the range from 47.5 to 70.9 cm, 3.31 to 5.45 g pot^−1^, 1.28 to 2.03 g pot^−1^ and 1.26 to 1.83 g pot^−1^, respectively (Table 1). Relative to the control, all treatments significantly improved the shoot and grain DW. However, except for CM 0.5%, the rest of the treatments significantly improved plant height and root DW, in comparison to the control treatment. The highest improvement in root and shoot DW by 58% and 64%, respectively, was observed in the BR 2% treatment. The BR 2% and BN 5% treatments showed the highest improvement in grain DW by 36% and 45% and plant height by 49% and 35%, respectively, relative to the control.

Likewise, the values of Chl a, Chl b, and RWC were in the range from 32.5 to 56 mg g^−1^ fresh weight (FW) and 26.9 to 48.5 mg g^−1^ FW and 60.7% to 78.8%, respectively (Table 1). All treatments significantly improved the Chl a and Chl b contents, with the exception of CM 0.5% only in case of Chl b, compared to the control. Likewise, with the exception of CM 0.5% and FeHP 2%, the rest of the treatments significantly improved the values of RWC compared to the control (Table 1). The BR 2% and BN 5% treatments showed the highest improvement in RWC values by 30% and 21%, and Chl b contents by 80% and 52%, respectively, relative to the control. Likewise, the highest improvement in Chl a contents was observed in BR 2%, and that was 72%, in comparison to the control.

### 2.3. Status of Micronutrients, Antinutrient and Biochemical Compounds in Pea Grain as Influenced by Amendments

**Figure 2:**
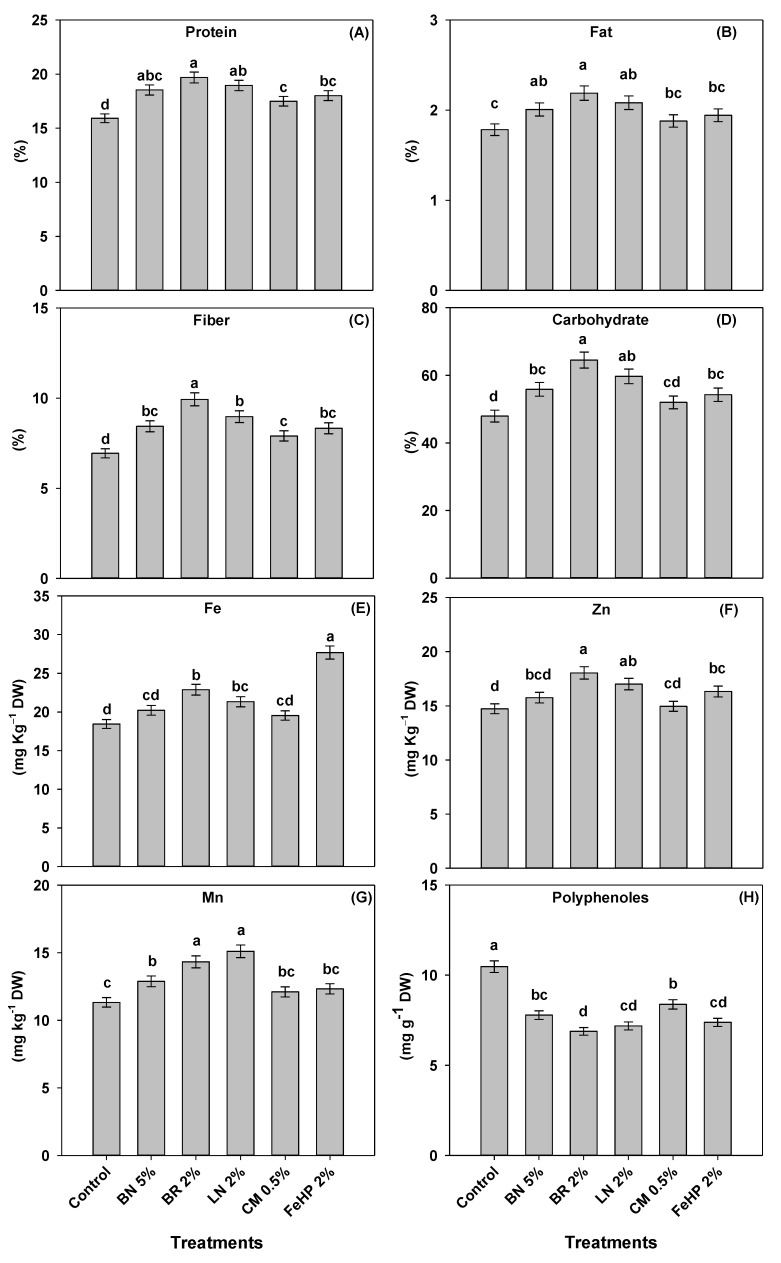
Effects of amendments in Pb-polluted soil on the contents of protein (**A**), fat (**B**), fiber (**C**) carbohydrate (**D**), and the concentrations of Fe (**E**), Zn (**F**), Mn (**G**) and polyphenols (**H**) in pea grain. Values are means of three replicates, error bars represent the standard error of the means, and the lower case alphabets indicate significant differences (*p* ≤ 0.05) among treatments based on one-way ANOVA (using LSD test, *p* = 0.05 at *df* = 5 and *n* = 3).

The data concerning protein, fat, fiber and carbohydrate contents in pea grain across treatments were in the ranges from 15.9% to 19.7%, 1.78% to 2.19%, 6.94% to 9.93% and 48% to 64.5%, respectively (Figure 2). With different exceptions, amending Pb-polluted soil with the selected amendments significantly improved the contents of grain biochemical compounds, compared to control. The highest improvement in protein contents by 16%, 24% and 19%, and fat contents by 13%, 23% and 17%, respectively, were observed in the BN 5%, BR 2% and LN 2% treatments. Similarly, BR 2% and LN 2% exhibited the highest significant improvement in grain carbohydrate contents by 34% and 24%, respectively, whereas fiber contents by 43% in BR 2%, relative to the control (Figure 2). 

The Fe, Zn and Mn concentrations in grain were in the ranges from 18.4 to 27.6 mg kg^−1^ DW, 14.7 to 18.0 mg kg^−1^ DW and 11.3 to 15.1 mg kg^−1^ DW respectively. The polyphenols were in the range 6.88 to 10.5 mg g^−1^ DW across all treatments (Figure 2). All amendments significantly decreased the grain polyphenol contents, while improvement in the grain Fe and Zn concentrations were found significant only in the BR 2%, LN 2% and FeHP 2% treatments. Likewise, only these BN 5%, BR 2% and LN 2% treatments significantly improved grain Mn concentrations (Figure 2). In this context, the BR 2%, LN 2% and FeHP 2% treatments reduced polyphenol contents up to 34%, 31%, and 29%, respectively, while the highest improvement in the grain Zn concentrations by 22% and 16% and Mn by 26% and 33% were observed in the BR 2% and LN 2% treatments, respectively. Likewise, the highest improvement in the grain Fe concentration by 50% was found in the FeHP 2% treatment.

### 2.4. Effect of Amendments on Antioxidant Defense Machinery and Oxidative Stress in Pea Plants

**Figure 3:**
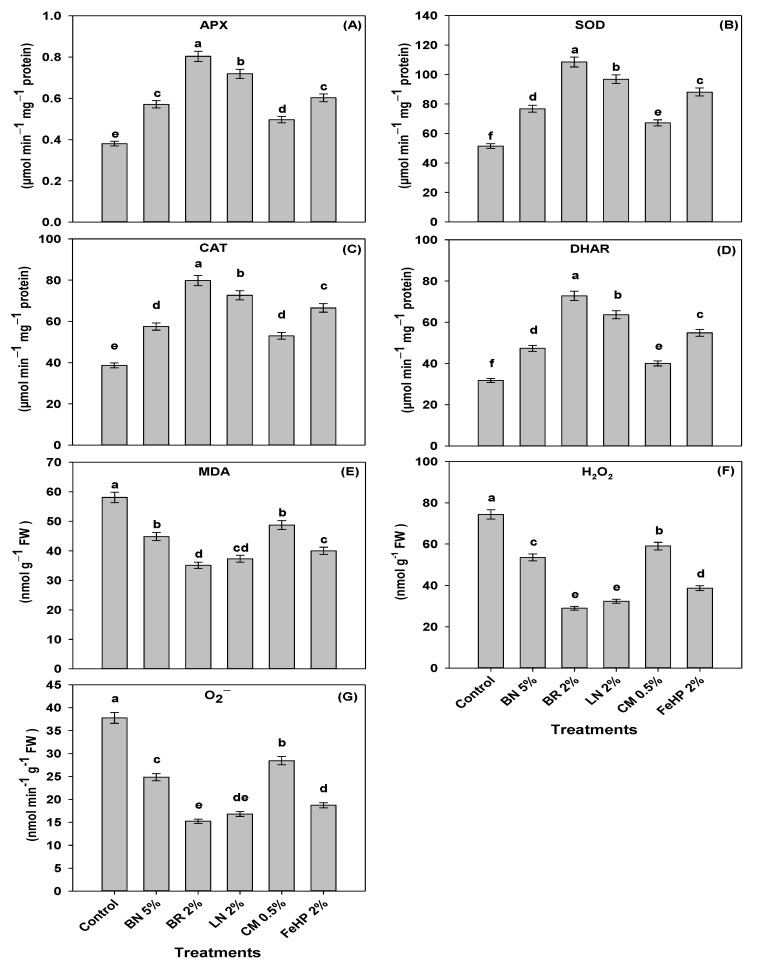
Effects of amendments inclusion in Pb-polluted soil on the activities of ascorbate peroxidase (APX) (**A**), superoxide dismutase (SOD) (**B**), catalase (CAT) (**C**) and dehydroascorbate reductase (DHAR) (**D**), as well as the contents of the malondialdehyde (MDA) (**E**), hydrogen peroxide (H_2_O_2_) (**F**) and O_2_^−^ generation rate (**G**) in pea leaves. Values are means of three replicates, error bars represent standard error of means, and the lower case alphabets indicate significant differences (*p* ≤ 0.05) among treatments based on one-way ANOVA (using LSD test, *p* = 0.05 at *df* = 5 and *n* = 3).

In all treatments, the activities of ascorbate peroxidase (APX), superoxide dismutase (SOD), catalase (CAT) and dehydroascorbate reductase (DHAR) were in the range from 0.38 to 0.80, 51.5 to 108.5, 38.7 to 53.0 and 31.8 to 72.9 µmol min^−1^ mg^−1^ protein, respectively (Figure 3). All treatments significantly improved the APX, SOD, CAT and DHAR activities, compared to the control. The topmost improvement in the APX, SOD, CAT and DHAR activities was found in BR 2% by 111%, 111%, 106% and 129%, respectively when compared to control.

The contents of malondialdehyde (MDA) and hydrogen peroxide (H_2_O_2_) were in the ranges from 35.1 to 58.1 and 29.0 to 74.4 nmol g^−1^ FW, while O_2_^−^ generation rate from 15.2 to 37.8 nmol min^−1^ g^−1^ FW, respectively (Figure 3). Interestingly, all treatments were capable of significantly reducing the contents of MDA and H_2_O_2_ as well as the O_2_^−^ generation rate, compared to the control. The obtained data for the BR 2% and LN 2% treatments showed the highest reduction in MDA contents up to 39% and 36%; H_2_O_2_ contents up to 61% and 56%, and O_2_^−^ generation rates up to 60% and 55%, respectively, compared to the control.

### 2.5. Principal Component Analysis and Pearson Coefficient Correlation (r^2^) Among Studied Attributes

**Figure 4:**
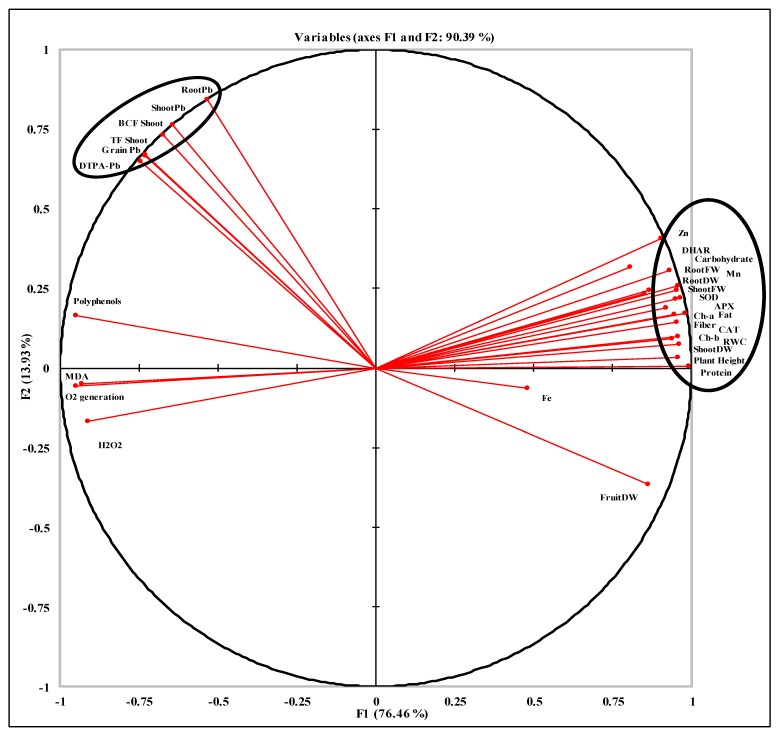
Principal component analysis (PCA) of studied attributes of pea grown under Pb stress with various soil amendments.

The values of Pearson correlation with their significance at probability levels (*p*) < 0.05, 0.01, 0.001 are presented in Table 2. It clearly shows that the plant biomass has positive significant correlation (*p* < 0.001) with studied physiological attributes like Chl a and Chl b, as well as antioxidant activities such as SOD, CAT, APX and DHAR. In addition, shoot dry weight is also positively significantly correlated (*p* < 0.001) with carbohydrates contents, total soluble proteins, Zn, fiber and fat contents of pea grain, as well as grain Mn (*p* < 0.01). However, shoot biomass was negatively correlated (*p* < 0.001) with the MDA and H_2_O_2_ contents, O_2_ generation and grain polyphenols. Moreover, the shoot dry weight has also negative significant correlation with BCF shoot, TF shoot, DTPA Pb, grain Pb and shoot Pb. Correlation studies presented in Figure 4 generated through PCA show that the studied attributes are categorized in two major groups. The first component of the PCA explained 76.6%, while the second component explained 13.93%, of the variance. The details of component loadings and communalities for each parameter is described in Tables S1 and S2, while the scree plot of PCA is represented in Figure S1

## 3. Discussion

### 3.1. Speciation of Pb in Pea Shoots, Roots, and Grain, Related BCF and TF Values and Pb Bioavailability in Post-Harvest Soil as Influenced by Amendments

All amendments noticeably reduced Pb concentration in pea roots, shoots, grain and bioavailable Pb (DTPA-extractable fraction), compared to control. The lowest Pb concentrations in roots, shoots, grain and DTPA-extract were found in BN 5% and CM, compared to control (Figure 1). Our results are in agreement with the results of previous studies, where the application of BN in Pb-polluted soil significantly reduced the concentrations of Pb in the shoots of pak choi and maize, as well as DTPA-extractable fraction [7]. The application of BN in Pb-polluted soil has also been reported to significantly reduce the Pb concentrations in rapeseed [5], rice grain and straw [1]. It has been reported that Pb concentrations were significantly reduced in the leachates after amending a Pb-polluted soil with CM [6]. The reduction in Pb concentrations in roots, shoots, grain and DTPA-extractable Pb fraction can be attributed to versatile characteristics of BN and CM. Application of CM in Pb-polluted soil reduces the bioavailability of Pb via three ways i.e., (1) sequestration of Pb by physical encapsulation by hydration products (struvite-K), (2) conversion of Pb into highly insoluble pyromorphite (Pb_5_(PO_4_)_3_X, X = Cl^−^, OH^−^, F^−^) and Pb-phosphate (Pb_3_(PO_4_)_2_) compounds and (3) raising soil pH after their application in the soil. It has been reported that pyromorphite and Pb-phosphate have extremely low solubility (Ksp ≅ 10^−60^−10^−85^ and 10^−6^, respectively) and thrive in extreme soil alkaline and acidic conditions [6,9]. 

Moreover, BN has vital characteristics that can alter the physicochemical characteristics of the soil. Application of BN increases soil pH which enhances sorption and precipitation of Pb in the soil. Furthermore, the larger surface of BN increases the CEC of the soil, which in turn reduces the Pb bioavailability to the plants [1,5,7].

The TF and BCF values for Pb were significantly reduced with all amendments, compared to the control. However, the least values of TF and BCF were observed after amending Pb-polluted soil with BN 5%, FeHP 2% and CM 0.5% (Table 1). The lower TF values of Pb are attributed to its accumulation in the cell membrane and vacuoles of the root, which is associated with the stable attribute of Pb in the soil-plant system [18]. The results of a previous study have explained that Pb is early recognized as a toxic compound by the roots of plants and is vacuolated either in the cell wall or vacuole, which in turn leads to its low translocation to the aerial parts [19]. The BCF stands for the transport potential of Pb from the soil to the plant body, and is dependent on the characteristics of soils and Pb speciation [20]. The BN 5%, FeHP 2% and CM 0.5% treatments showed the least BCF values of Pb, compared to the control. These lower BCF values of Pb indicate a very low transfer of Pb in pea showing its least bioavailability in the BN 5%, FeHP 2% and CM 0.5% amended soil, compared to control treatment [21].

### 3.2. Agronomic, Photosynthetic and Biophysical Parameters of Pea Plant as Influenced by Amendments

With few exceptions, all treatments significantly improved shoot, root and grain DW, as well as plant height, RWC and chlorophyll contents in pea, compared to control. However, BR treatment showed the highest shoot and root DW, and Chl-a contents, while BN and BR treatments showed highest grain DW, plant height, Chl-b and RWC contents, compared to control (Table 1). An improvement in shoot, root and grain DW, as well as plant height of brinjal [13], the contents of Chl-a and Chl-b in menthol [3] and RWC in chicory were observed after BR application in Pb-polluted soil [22]. Likewise, the application of BN in Pb-polluted soil significantly improved shoot DW and Chl contents in Chinese cabbage [7]. Improvement in the plant DW, RWC and Chl contents could be attributed to the various characteristics of BR like provision of essential nutrients, improvement in WHC and alteration in redox condition of the soil [23,24,25]. Likewise, improvement in the agronomic and biophysical parameters of plants is also due to the alleviation of Pb toxicity to them after the Pb has been immobilized onto the BR surface [3,12,13]. Moreover, improvement in soil CEC after the application of BN is responsible for the reduced phytoavailability of Pb. It has been reported that improvement in plant biomass and associated parameters are mainly due to the alleviation of Pb toxicity to the plants [7,26].

### 3.3. Status of Micronutrients, Antinutrient and Biochemical Compounds in Pea Grain as Influenced by Amendments

With few exceptions, the application of all amendments significantly improved the contents of protein, fat, fiber and carbohydrates, while it decreased the contents of polyphenols, compared to control. However, the best results regarding these parameters were observed in BR and LN treatments (Figure 2). It has been reported that Pb toxicity reduced the contents of biochemical compounds while there was an increase in the polyphenol contents in brinjal [13], sesame [27] and *Conocarpus erectus* [10]. The results of our study are in line with the findings of previous investigations where improvement in the contents of biochemical compounds and reduction in polyphenol contents of different plants grown in BR amended metal-polluted soils was reported [13,27]. Likewise, BR application in Pb-polluted soil improved the biochemical compounds of spinach [14]. Improvement in the contents of biochemical compounds in pea grain is due to the vital characteristics of BR and LN. Biochar and LN have very high WHC, and are rich in organic matter. Application of BR and LN in soil improves the activities of rhizosphere microbes due to the decomposition of these organic materials and the enhanced release of dissolved organic carbon, which in turn are responsible for enhancing the plant protein contents [15,27]. Amending soil with BR and LN increases the WHC of soil and improves the availability of water to the plants through xylem, and augments the metabolic activity in the plant. Improvement in the plant metabolic activity enhances the plant biochemical compounds and reduces polyphenol contents [15,16]. 

Enhancement in the contents of biochemical compounds could also be associated with the alleviation of Pb toxicity to the plants [10,14]. Application of LN in soil improves the soil organic matter content. The hydroxyl and carboxyl groups present on organic matter react with OH^−^ in soil and make them electronegative. This special feature of LN increases the variable negative charges in the soil which promote the adsorption of Pb ions on the soil colloids, thereby reducing Pb toxicity to the plants [15,16]. Likewise, BR also alleviates Pb toxicity to the plants via adsorbing Pb ions onto the larger surface area [13,14].

In our experiment, the highest grain Mn and Zn concentrations were found with BR 2% and LN 2% treatments, compared to control. Likewise, the highest significant Fe concentration was observed with FeHP treatment, compared to control (Figure 2). It has been reported that amending metal-polluted soil with BR significantly improved the concentrations of Mn and Zn in brinjal [13] and sunflower [28]. Likewise, an improvement in the Fe concentrations of rice grain was reported after the application of iron compounds [29]. The higher concentrations of Fe in pea grain could be attributed to the presence of Fe in the FeHP amendment. Furthermore, BR application in the soil also provides essential nutrients to the plants and increases the concentrations of Zn and Mn [16]. Similarly, LN contains several functional groups that help to strongly adsorb Pb ions which reduce the Pb bioavailability in the soil [15,16]. Therefore, the reduced bioavailability of Pb in the soil improved the phyto-availability of micronutrients, especially Mn and Zn and their uptake by the plant roots due to their antagonistic behavior with Pb ions [30]. It has been reported that the sum of released cations like K, Na, Ca and Mg from amendments is almost equal to the quantity of adsorbed metal ions, explaining the primary function of cation exchange in Pb sorption by BH [31].

### 3.4. Status of Antioxidant Defense Machinery and Oxidative Stress in Pea Plant as Influenced by Amendments

In our experiment, all of the amendments noticeably reduced the MDA, H_2_O_2_ contents and the O_2_^−^ generation rate, as well as improved the activities of APX, SOD, CAT and DHAR in pea plant relative to the control treatment. However, the most pronounced results regarding these parameters were found in BR treatment followed by LN treatment (Figure 3). Our results are in line with the findings of previous studies where the activities of SOD, CAT, peroxidase (PER), ascorbic acid (AsA) and APX in brinjal [15], SOD, CAT and PER in *Mentha arvensis* and CAT, PER and polyphenol oxidase (PPO) in chicory [22], were enhanced with the incorporation of BR in Pb-polluted soil. Whereas, the contents of the H_2_O_2_ and O_2_^−^ generation rate were significantly reduced in chicory with BR application in a Pb-polluted soil [22]. Plants produce antioxidant enzymes like APX, SOD, CAT and DHAR upon the exposure to heavy metals stress which acts as a defense system against oxidative stress; the latter is the higher production of ROS as a result of oxidizing chain reactions [16]. The mechanism responsible for this improvement in antioxidant activities and the reduction of ROS contents in the plants is due to the fundamental characteristics of BR; i.e., promotion of plant health, provision of essential nutrients, resistance against metal stress after they get immobilized on BR, having the larger surface area and high cation exchange capacity [16]. Similarly, LN adsorbs metal ions [15] and decreases the metal stress to the plant, which in turn increases the mobility of essential nutrients to the plant. Higher mobility of essential nutrients to plants is known to improve their vigor and thereby reduce the contents of reactive oxygen species in them [16].

## 4. Materials and Methods

### 4.1. Collection of Experiment Soil and its Characterization

Experimental soil was purchased from a plant shop named “Evergreen nursery, Faisalabad, Pakistan”. The soil was air-dried and sieved through a 2 mm sieve to remove stones and debris. The physicochemical properties of the soil were determined by employing standard methods. Soil pH was determined by the method of McLean, [32] by preparing a suspension (soil:deionized water, 1:1) followed by shaking for approximately 1 h and measurement on a calibrated pH meter (model WTW7110, Weilheim, Germany). Similarly, soil texture was determined using the hydrometer method, soil organic matter using the Walkley-Black method and Cation exchange capacity (CEC) was determined by the approaches of Gee and Bauder, [33], Jackson [34], and Rhoades [35], respectively. Determination of total phosphorus, exchangeable potassium and calcium carbonate, were carried out according to the methods of Watanabe and Olsen [36], Richards [37] and Allison and Moodie [38], respectively. Likewise, the bioavailable fraction of Pb in the Pb-polluted soil was measured on the atomic absorption spectrophotometer (AAS, PerkinElmer AAnalyst™ 800, Shelton, CT, USA) after extracting the soil with 5 mM diethylenetriaminepentaacetic acid (DTPA extractant (soil to DTPA extractant, 1:2) [39]. The properties of experimental soil are presented in Table 3.

### 4.2. Soil Spiking with Pb

Soil was amended with Pb(NO_3_)_2_ to obtain a 1000 mg kg^−1^ Pb concentration in the soil. For this purpose, a known amount of Pb(NO_3_)_2_ was dissolved in distilled water. Later, this Pb(NO_3_)_2_ solution was poured in the soil and properly mixed. The soil was homogenized and packed in plastic sacks that were later kept at 25 °C for 60 days in a dark room. The soil in plastic sacks was manually mixed with a spatula twice a week for a homogeneous distribution of moisture. During this incubation period, distilled water was used to maintain the moisture at 65% water holding capacity (WHC) of the soil. The soil was air-dried after the end of the incubation. 

### 4.3. Addition of Immobilizing Agents

Five reducing agents, including BN, BR, LN, CM 0.5% and FeHP were used for the immobilization of Pb in Pb-spiked soil. The BN was acquired from Jinan Yuansheng Chemical Technology Co., Ltd., Licheng District, Jinan, Shandong, China. The preparation and characterization of BR used in this experiment have been described in Shahbaz et al. [23]. Lignin was procured from the Jinan Yuansheng Chemical Technology Co., Ltd., Licheng District, Jinan, Shandong, China and CM from a local store. Likewise, a mixture of [Sodium phosphate (1000 mL, 0.5 mol L^−1^) after a reaction with ferric chloride (1000 mL, 0.3 mol L^−1^)] was prepared and placed in a hermetic container at 35 °C for 24 h. The pH of the aforementioned reaction mixture was fixed at 4, using 1 M NaOH solution. After the completion of the reaction, the pH of the reaction mixture rose to 4.3. Ultimately, the resultant product i.e., (FeHP), was wiped out with distilled water and desiccated at 65 °C for 15 h. 

### 4.4. Pot Experiment

Overall, the pot experiment consisted of six treatments i.e., BN, BR, LN, CM and FeHP and control Pb-spiked soil without any amendment (Table 4). The percentage of each immobilizing amendment used in this study was selected after carefully reviewing the previous studies i.e., BR 2% [40], BN 5% [1], LN 2% [15], CM 0.5% [6] and FeHP 2% [17], respectively. The resulting uniform mixture was incorporated with leftover soil via mechanical shaker, while maintaining the moisture at 65% WHC followed by incubation at 25 °C for six weeks in darkness. After the incubation, the treated soil was transferred to the plastic pots (height 33 cm, diameter 25.4 cm). The relocation of the experiment was accomplished in the field area of Government College University Faisalabad, Faisalabad, Pakistan with intense care, considering the appropriate environmental conditions, for instance, suitable temperature 25 °C, proper light 8–10 h and moisture 50%. 

Pots were moistened to achieve suitable sowing conditions. Pea seeds were procured from Ayub Agriculture Research Institute, Faisalabad, Pakistan and soaked in water for 8 h preceding sowing in the pots. Eight seeds were sown per pot, which germinated in about 10 days within sowing. The pea plants were fertilized using balanced plant fertilizer [Grow Fertilizer (18-18-18), White Flower Farm, Litchfield, CT, USA] at the age of three weeks. Some parameters like plant height, length of plant and biomass (root and shoot), were assessed by using a portable measuring stick after 60 days of pea plant growth. After 60 d of growth, plants were harvested at the base and divided into root and shoot parts.

### 4.5. Plant and Soil Analysis

#### 4.5.1. Estimating the Pb Concentrations in Plant Parts and DTPA Extract

The harvested soil was extracted from the pot with intense care, air-dried and sieved through a 2 mm sieve. An aliquot of harvested soil was used to measure its pH, as described earlier.

DTPA-extractable Pb was extracted by using the standard methodology of Lindsay and Norvell, [39], and the extracts were analyzed on AAS. Subsequently, plants (root and shoot) were rinsed to remove the adhered dust and dirt. The pea plant biomass was desiccated in an oven (Memmert, Beschickung-loading, model 100–800, Schwabach, Germany) at 70 °C for 24 h to get constant dry weight. The dried plant material was pulverized in a grinder (IKAWerke, MF 10 Basic, Staufen, Germany) followed by di-acid digestion (HNO_3_:HClO_4_ = 2:1) after sieving at 0.5mm as devised by Jones and Case [41]. Eventually, the Pb concentration in plant digest was analyzed on AAS.

#### 4.5.2. Chlorophyll Contents, Antioxidant Enzymes Activities and Reactive Oxygen Species (ROS) Contents in Barley Leaf

The chlorophyll a (Chl-a) and chlorophyll b (Chl-b) contents in the leaves were assessed following the methods of Hiscox and Israelstam [42]. To this end, 1 g of fresh leaf sample was homogenized in 20 mL of methanol, chloroform and water (12:5:3 ratio). The contents of Chl-a and Chl-b were assessed by measuring the absorbance on a spectrophotometer at 664.5 and 647.4 nm, respectively. 

The antioxidant enzymes [SOD, CAT, APX and DHAR] in pea plant were determined by the methodology of El-Shabrawi et al. [43], Aebi [44], Nakano and Asada [45], respectively. The reaction mixture for SOD was prepared by mixing supernatant (1 mL) with potassium phosphate (K-P) buffer (50 mM), CAT (0.1 u), nitro blue tetrazolium (NBT, 2.24 mM), xanthine (2.36 mM, pH 7) and xanthine oxidase (0.1 u), as it follows the xanthine–xanthine oxidase system. The mixture for APX was prepared by mixing the supernatant (0.5 mL) with [AsA (0.5 mM), H_2_O_2_ (0.1 mM), sodium phosphate buffer (pH 7) and ethylenediaminetetraacetic acid (EDTA) (0.25 mL)]. The [dehydroascorbate (DHA, 0.1 mM), glutathione (GSH, 2.5 mM) and K-P buffer (50 mM, pH 7)] were mixed with supernatant [0.5 mL] for the mixture of DHAR. Similarly, the CAT reaction was commenced with H_2_O_2_ and its degradation was analyzed. Finally, the variations in absorbance were recorded via spectrophotometer at 560 nm, 240 nm, 290 nm and 265 nm for SOD, CAT, APX and DHAR, respectively. The specific activity was observed for the extraction coefficient at 40 mM cm to obtain CAT and extinction coefficient at 2.8 mM cm and 14 mM cm to obtain APX and DHAR. 

The contents of MDA, H_2_O_2_ and O_2_^−^ generation rate were measured by the methods of Jambunathan, [46], Velikova et al. [47] and Yang et al. [48], respectively. The reaction mixtures were prepared with fresh leaf tissue (500 mg) and assimilated with trichloroacetic acid (TCA, 5 mL, 0.1%) for MDA, K-P buffer [(5 mL) for H_2_O_2_ and (12 mL, 65 mM, pH 7.8)] for O_2_. Afterward, the mixtures were centrifuged at 10,000× *g* for 15 min and supernatants (2.5 mL, 0.5 mL & 5 mL) for MDA, H_2_O_2_ and O_2_, respectively, were used. Subsequently, supernatants were assorted with thiobarbituric acid (TBA, 1 mL, 0.5% *w*/*v*) in TCA (20%), heated at 95 °C for 30 min and later chilled in an ice bath for MDA. Similarly, the mixture (TCA, 5 mL, 0.1% *w/v*) was prepared in K-P buffer (10 mM, pH 7) using potassium iodide (1 M, 1 mL) for H_2_O_2_ determination. For the estimation of O_2_^−^ generation, K-P buffer (0.9 mL, 65 mM, pH 7.8) was mixed with hydroxylamine hydrochloride (1 mL, 10 mM), sulfanilamide (1 mL, 17 mM) and naphthylamine (1 mL, 7 mM) before incubation at 25 °C for 20 min. The variation in absorbance was deliberated via Beer and Lambert’s equation to calculate MDA at 532 nm–600 nm. While the absorbance for the H_2_O_2_ and O_2_^−^ generation rate were scrutinized via spectrophotometer at 390 nm and 530 nm, respectively [49].

#### 4.5.3. Estimation of grain Biochemical Compounds and Micronutrients

Various standard protocols were used to analyze the protein, fat, fiber, carbohydrate and polyphenols in the ground pea grain samples. A standard approach was developed for the estimation of plant protein by using the protein dye-binding method involving an equal volume of sample buffer (impeded by Bradford assay) added into the protein reagent to compensate for the interference. Bovine serum albumin was used to accomplish the reaction [50]. The association of official analytical chemists (AOAC) [51] methods were used for the estimation of fat, fiber and carbohydrates in pea grain. 

Polyphenols in pea grain were determined by using the Folin-Ciocalteu method proposed by Singleton et al. [52]. Following this method, the calibration curve of the standard for gallic acid was plotted for the estimation of phenolic compounds in pea grain, and represented as mg g^−1^ equivalent to gallic acid (GAE) after recording the absorbance at 760 nm on a spectrophotometer. 

Some of the pea grain were crushed in a grinder (IKAWerke, MF 10 Basic, Staufen, Germany) followed by sieving at 0.5 mm. The grounded grains were subjected to di-acid (HNO_3_:HClO_4_, 2:1) digestion. Afterwards, further characterizations of pea grain micronutrients (Ca, Zn, Fe, Mg, Mn, Ni) were performed spectrophotometrically (PerkinElmer, AAnalyst 100, Waltham, MA, USA). The aforementioned process was developed by Jones and Case [41]. 

#### 4.5.4. Computation of Translocation Factor (TF) and Bioconcentration Factor (BCF) of Pb

The values of BCF and TF of Pb were calculated by Equations (1) and (2) respectively as recommended by Salazar and Pignata [53].

BCF = *C*shoot/*C*soil (1)

TF = *C*shoot/*C*root (2)

where, the *C*shoot, *C*root and *C*soil are the concentrations of Pb in the shoots (mg kg^−1^ DW), roots (mg kg^−1^ DW) and soil (mg kg^−1^ DW soil), respectively.

### 4.6. Statistical Analysis

A completely randomized design was used to execute this pot experiment, and the results were interpreted by using one-way Analysis of Variance (ANOVA) with the help of the Statistix 8.1 software package (Copyright 2005, Analytical software, Tallahassee, FL, USA). The described means are the average of three replicates, and are stated with their standard error (SE). A least significant difference (LSD) test was carried out to detect the significant difference (*p* < 0.05) between treatment means. The principle component analysis (PCA) and Pearson coefficient correlation (r^2^) among studied attributes was computed by using the xlstat software version 4.15 (Addinsoft, Paris, France). The table of communalities for each parameter was generated with the IBM SPSS Statistics software windows version 25 (IBM Corp, Armonk, NY, USA).

## 5. Conclusions

In this pot experiment, Pb-polluted soil was amended with different amendments i.e., BC 2%, BN 5%, LN 2%, CM 0.5% and FeHP 2%, and their effects on different parameters of pea were observed. Results showed that BN 5%, FeHP 2% and CM 0.5% significantly reduced the concentrations of Pb in shoots, roots and grain, as well as the TF and BCF values of Pb in the order FeHP 2% > CM 0.5% > BN 5%. The agronomic (plant height, shoot, root and grain DW), biochemical (chl a and chl b contents and grain biochemistry), biophysical (RWC) parameters and grain micronutrient concentrations were improved with BR 2%. However, the 2% BR rate that we used in our pot experiment may not be economically viable for field scale application.

## Figures and Tables

**Table 1:** Influences of bentonite (BN), biochar (BR), lignin (LN), cement (CM) and iron hydroxyl phosphate (FeHP) on growth and biomass, chlorophyll contents and relative water content (RWC) in leaves of pea, translocation factor (TF) and the bioconcentration factor (BCF) values for Pb in plant and Pb contents in shoot, root and grain grown on a Pb-contaminated soil are illustrated. Numerical values represent means (from three replicates i.e., *n* = 3) along bars sharing identical alphabetic letters which are statistically (at *p* < 0.05) non-significant to each other. These values are mean of three replicates ± SE.

Treatments	Growth Parameters					Chlorophyll Contents		Pb Translocation		Pb Contents		
	Shoot DW (g pot^−1^)	Root DW (g pot^−1^)	Grain DW (g pot^−1^)	Plant Height (cm plant^−1^)	RWC (%)	Chl a (mg g^−1^ FW)	Chl b (mg g^−1^ FW)	TF	BCF	Shoot (mg kg^−1^)	Root (mg kg^−1^)	Grain (mg kg^−1^)
Control	3.3 ± 0.1 e	1.3 ± 0.0 e	1.3 ± 0.0 d	47.5 ± 1.7 d	60.7 ± 2.2 d	32.5 ± 1.2 e	26.9 ± 1.0 d	0.432 ± 0.02 a	0.37 ± 0.01 a	1.21 ± 0.02 b	1.08 ± 0.02 cd	0.16 ± 0.001 a
BN 5%	4.7 ± 0.2 b	1.7 ± 0.1 b	1.8 ± 0.1 ab	64.3 ± 2.3 ab	73.4 ± 2.7 ab	47.5 ± 1.7 b	40.9 ± 1.5 ab	0.339 ± 0.01 d	0.22 ± 0.01 d	1.04 ± 0.02 c	1.13 ± 0.02 c	0.10 ± 0.001 d
BR 2%	5.4 ± 0.2 a	2.0 ± 0.1 a	1.7 ± 0.1 a	70.9 ± 2.6 a	78.8 ± 2.9 a	56.0 ± 2.0 a	48.5 ± 1.8 a	0.361 ± 0.01 bc	0.27 ± 0.01 bc	1.45 ± 0.03 a	1.49 ± 0.03 a	0.11 ± 0.001 c
LN 2%	4.4 ± 0.2 bc	1.6 ± 0.1 be	1.6 ± 0.1 bc	59.8 ± 2.2 bc	69.6 ± 2.5 bc	44.0 ± 1.6 bc	38.6 ± 1.4 bc	0.379 ± 0.01 b	0.28 ± 0.01 b	1.24 ± 0.02 b	1.21 ± 0.02 b	0.12 ± 0.001 b
CM 0.5%	3.8 ± 0.1 d	1.4 ± 0.1 de	1.5 ± 0.1 c	53.4 ± 1.9 cd	64.0 ± 2.3 cd	37.6 ± 1.4 d	30.4 ± 1.1 d	0.360 ± 0.01 cd	0.24 ± 0.01 cd	0.94 ± 0.02 d	0.95 ± 0.02 e	0.09 ± 0.001 e
FeHp 2%	4.2 ± 0.2 cd	1.5 ± 0.1 cd	1.6 ± 0.1 bc	57.4 ± 2.1 c	67.1 ± 2.4 bcd	41.8 ± 1.5 cd	35.1 ± 1.3 c	0.371 ± 0.01 bcd	0.26 ± 0.01 bcd	1.08 ± 0.02 c	1.04 ± 0.02 d	0.10 ± 0.001 d

**Table 2:** Pearson coefficient correlation (r^2^) values of studied attributes of pea showing significance differences grown under Pb stress with various soil amendments.

	SDW	GDW	Chl-a	Chl-b	RWC	DTPA-Pb	S-Pb	G-Pb	TF-S	BCF-S	Prot	Carb	Fat	Fiber	Mn	Zn	PPs	APX	CAT	SOD	DHAR	MDA	H_2_O_2_	O_2_-ge
SDW	1.000											5												
GDW	0.852 ***	1.000																						
Chl-a	0.999 ***	0.845 ***	1.000																					
Chl-b	0.994 ***	0.841 ***	0.996 ***	1.000																				
RWC	0.994 ***	0.860 ***	0.996 ***	0.995 ***	1.000																			
DTPA-Pb	−0.634 **	−0.837 ***	−0.613 **	−0.581 **	−0.593 **	1.000																		
S-Pb	−0.573 *	−0.839 ***	−0.552 *	−0.513 *	−0.546 *	0.973 ***	1.000																	
G-Pb	−0.631 **	−0.832 ***	−0.611 **	−0.573 *	−0.590 *	0.998 ***	0.981 ***	1.000																
TF-S	−0.684 **	−0.894 ***	−0.665 **	−0.630 **	−0.663 **	0.965 ***	0.986 ***	0.972 ***	1.000															
BCF-S	−0.590 **	−0.840 ***	−0.568 *	−0.533 *	−0.561 *	0.982 ***	0.995 ***	0.985 ***	0.988 ***	1.000														
Prot	0.940 ***	0.852 ***	0.934 ***	0.939 ***	0.920 ***	−0.743 ***	−0.636 **	−0.721 ***	−0.723 ***	−0.674 **	1.000													
Carb	0.939 ***	0.720 ***	0.939 ***	0.949 ***	0.922 ***	−0.553 *	−0.426 ns	−0.531 *	−0.539 *	−0.470 *	0.964 ***	1.000												
Fat	0.949 ***	0.766 ***	0.949 ***	0.963 ***	0.939 ***	−0.576 **	−0.460 *	−0.554 *	−0.572 *	−0.502 *	0.974 ***	0.996 ***	1.000											
Fiber	0.949 ***	0.751 ***	0.947 ***	0.948 ***	0.923 ***	−0.629 **	−0.504 *	−0.612 **	−0.605 **	−0.542 *	0.976 ***	0.992 ***	0.987 ***	1.000										
Mn	0.723 ***	0.569 *	0.722 ***	0.762 ***	0.717 ***	−0.434 ns	−0.280 ns	−0.386 ns	−0.379 ns	−0.353 ns	0.864 ***	0.896 ***	0.899 ***	0.856 ***	1.000									
Zn	0.867 ***	0.603 **	0.874 ***	0.891 ***	0.845 ***	−0.422 ns	−0.266 ns	−0.397 ns	−0.367 ns	−0.302 ns	0.894 ***	0.960 ***	0.948 ***	0.952 ***	0.853 ***	1.000								
PPs	−0.826 ***	−0.827 ***	−0.818 ***	−0.815 ***	−0.786 ***	0.846 ***	0.724 ***	0.823 ***	0.764 ***	0.754 ***	−0.947 ***	−0.860 ***	−0.868 ***	−0.901 ***	−0.771 ***	−0.823 ***	1.000							
APX	0.891 ***	0.691 **	0.891 ***	0.904 ***	0.863 ***	−0.576 **	−0.423 ns	−0.548 *	−0.517 *	−0.468 *	0.960 ***	0.983 ***	0.976 ***	0.984 ***	0.904 ***	0.976 ***	−0.906 ***	1.000						
CAT	0.850 ***	0.684 **	0.849 ***	0.857 ***	0.811 ***	−0.633 **	−0.471 *	−0.606 **	−0.542 *	−0.510 *	0.944 ***	0.944 ***	0.935 ***	0.961 ***	0.856 ***	0.958 ***	−0.943 ***	0.986 ***	1.000					
SOD	0.867 ***	0.685 **	0.868 ***	0.878 ***	0.833 ***	−0.594 **	−0.435 ns	−0.567 *	−0.514 *	−0.472 *	0.942 ***	0.955 ***	0.947 ***	0.966 ***	0.860 ***	0.975 ***	−0.926 ***	0.991 ***	0.997 ***	1.000				
DHAR	0.864 ***	0.642 **	0.867 ***	0.882 ***	0.835 ***	−0.518 *	−0.356 ns	−0.491 *	−0.446 ns	−0.396 ns	0.927 ***	0.965 ***	0.954 ***	0.966 ***	0.877 ***	0.991 ***	−0.885 ***	0.993 ***	0.987 ***	0.995 ***	1.000			
MDA	−0.833 ***	−0.741***	−0.831 ***	−0.839 ***	−0.796 ***	0.706 ***	0.554 *	0.677 **	0.610 **	0.590 **	−0.948 ***	−0.913 ***	−0.912 ***	−0.937 ***	−0.839 ***	−0.919 ***	0.975 ***	−0.964 ***	−0.990 ***	-0.983 ***	-0.961 ***	1.000		
H_2_O_2_	−0.787 ***	−0.648 **	−0.787 ***	−0.801 ***	−0.747 ***	0.612 **	0.440 ns	0.580 **	0.497 *	0.479 *	−0.909 ***	−0.902 ***	−0.894 ***	−0.920 ***	−0.847 ***	−0.939 ***	0.938 ***	−0.965 ***	−0.992 ***	-0.987 ***	-0.973 ***	0.990 ***	1.000	
O_2_-ge	−0.804 ***	−0.722 ***	−0.802 ***	−0.811 ***	−0.765 ***	0.700 ***	0.544 *	0.671 **	0.593 **	0.578 **	−0.928 ***	−0.889 ***	−0.888 ***	−0.916 ***	−0.821 ***	−0.908 ***	0.972 ***	−0.951 ***	−0.985 ***	-0.976 ***	-0.952 ***	0.998 ***	0.992 ***	1.000

*, ** and *** = significant at 0.05, 0.01, 0.001 levels respectively; ns = non-significant. SDW: Shoot dry weight; GDW; Grain dry weight; GDW: Chl-a: Chlorophyll a contents; Chl-b: Chlorophyll b contents; RWC: Relative water contents; R-Pb: Root Pb contents; S-Pb: Shoot Pb contents; G-Pb: Grain Pb contents; TF-S: TF shoot; BCF-S: BCF-Shoot; Prot: Protein; Carb: Carbohydrates; PP: Polyphenols; O2-ge: O2 generation.

**Table 3:** Physiochemical characteristics of experimental soil.

Characteristics	Units	Amount
Clay	%	29.7 ± 1.07
Silt	%	27 ± 0.97
Sand	%	40.3 ± 0.80
Organic matter content (OMC)	%	0.84 ± 0.03
Bicarbonate (HCO3)	%	0.17 ± 0.01
pH	-	8.4 ± 0.30
Cation exchange capacity (CEC)	cmolc kg^−1^	29.2 ± 1.06
Electrical conductivity (EC)	DSm^−1^	3.8 ± 0.14
Content of calcium carbonate (CaCO3)	%	2.9 ± 0.11
Phosphorus (P)	mg kg^−1^	8.3 ± 0.30
Potassium (K)	mg kg^−1^	81 ± 2.94
Nitrogen (N)	mg kg^−1^	174 ± 6.31
Total Pb	mg kg^−1^	1000 ± 36.2
diethylenetriaminepentaacetic acid (DTPA)-extractable Pb	mg kg^−1^	6.1 ± 0.22

**Table 4:** Overview of treatment plan considered in this pot experiment using BN = Bentonite, BR = Biochar, LN = Lignin CM = Cement and FeHP = Iron hydroxyl phosphate.

Treatments	Abbreviations	Input Amounts of Both Amendments (g pot^−1^)
Control	Control	-
Bentonite (5%)	BN	150
Biochar (2%)	BR	60
Lignin (2%)	LN	60
Cement (0.5%)	CM	15
Iron Hydroxyl phosphate (2%)	FeHP	60

## Supplementary Materials

The following are available online at https://www.mdpi.com/2223-7747/8/12/571/s1, Table S1: Factor loadings for PCA (Principle component analysis) of studied attributes of pea grown under Pb stress with various soil amendments. Table S2: Communalities of studied attributes of pea grown under Pb stress with various soil amendments. Figure S1: Scree plot representing the Eigen values and cumulative variability (%) in relation to factors used in PCA
