# Supplementary material for: Bentonite and Biochar Mitigate Pb Toxicity in Pisum sativum by Reducing Plant Oxidative Stress and Pb Translocation
Source: Plants (Basel). 2019 Dec 5;8(12):571. doi: 10.3390/plants8120571 (PMC6963978; doi:10.3390/plants8120571)
Supplement: Supplementary file 1 [file plants-08-00571-s001.pdf]

Table S1. Factor Loadings for PCA (principle component analysis) of studied attributes of pea grown under Pb stress with various soil amendments.

	F1	F2	F3	F4	F5
Plant Height	0.958	0.032	–0.267	0.080	0.053
Shoot DW	0.960	0.073	–0.258	0.085	–0.005
Root DW	0.921	0.189	–0.327	0.095	–0.014
Fruit DW	0.862	–0.366	–0.195	0.036	0.290
Shoot FW	0.852	0.233	–0.427	0.191	–0.028
Root FW	0.864	0.245	–0.367	0.165	–0.177
Chl-a	0.956	0.097	–0.258	0.100	0.012
Chl-b	0.954	0.145	–0.242	0.064	0.077
Relative Water Contents	0.937	0.093	–0.319	0.074	0.076
Root Pb	–0.533	0.844	–0.010	–0.055	0.017
DTPA Pb	–0.746	0.650	–0.112	0.066	0.069
Shoot Pb	–0.644	0.763	0.047	0.021	0.016
Fruit Pb	–0.731	0.671	–0.074	0.021	0.099
TF Shoot	–0.729	0.667	0.146	0.049	0.018
BCF Shoot	–0.672	0.732	0.029	0.106	0.029
Total Protein	0.992	0.007	0.007	–0.126	0.021
Grain Carbohydrate	0.957	0.257	–0.041	–0.113	–0.051
Grain Fat	0.966	0.220	–0.065	–0.121	0.020
Grain Fiber	0.979	0.170	–0.004	–0.051	–0.101
Grain Mn^{2+}	0.807	0.316	0.134	–0.468	0.112
Grain Zn^{2+}	0.903	0.406	0.130	0.045	–0.010
Grain Fe^{3+}	0.481	–0.065	0.638	0.594	0.071
Grain Polyphenols	–0.949	0.163	–0.267	0.043	–0.009
APX activity	0.954	0.246	0.139	–0.089	–0.039
Catalase activity	0.947	0.168	0.267	–0.021	–0.060
Super Oxide Dismutase	0.948	0.216	0.231	0.004	–0.026
DHAR activity	0.930	0.308	0.196	–0.013	–0.029
MDA contents	–0.949	–0.057	–0.308	0.026	–0.018
H_2O_2 contents	–0.911	–0.169	–0.377	0.019	0.000
O_2 generation	–0.931	–0.051	–0.359	0.005	–0.027

Table S2. Communalities of studied attributes of pea grown under Pb stress with various soil amendments.

Communalities		
	**Initial**	**Extraction**
Shoot FW	1.000	0.963
Shoot DW	1.000	0.993
Plant Height	1.000	0.991
Root DW	1.000	0.991
Fruit DW	1.000	0.915
Root FW	1.000	0.941
Chl a	1.000	0.990
Chl b	1.000	0.990
Relative Water Content	1.000	0.989
Root Pb	1.000	0.997
DTPA Pb	1.000	0.991
Shoot Pb	1.000	0.999
Fruit Pb	1.000	0.990
TF Shoot	1.000	0.997
BCF Shoot	1.000	0.988
Total Protein	1.000	0.984
Grain Carbohydrate	1.000	0.985
Grain Fat	1.000	0.985
Grain Fiber	1.000	0.987
Grain Mn^{2+}	1.000	0.769
Grain Zn^{2+}	1.000	0.998
Grain Fe^{3+}	1.000	0.642
Grain Polyphenols	1.000	0.998
APX	1.000	0.991
CAT	1.000	0.996
SOD	1.000	0.999
DHAR	1.000	0.999
MDA	1.000	0.999
H_2O_2	1.000	1.000
O_2 generation	1.000	0.999
Extraction Method: Principal Component Analysis.		

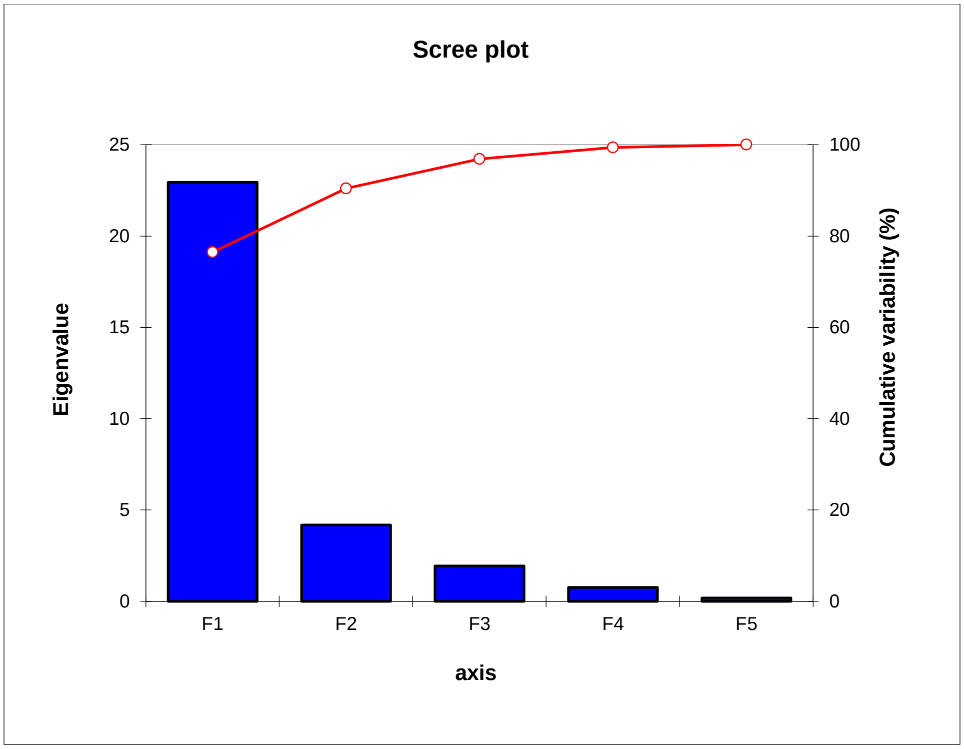

Figure S1 Scree Plot representing the Eigenvalues and cumulative variability (%) in relation to factors used in PCA
